# Blood's Concentration of Lead and Arsenic Associated with Anemia in Peruvian Children

**DOI:** 10.1155/2021/7283514

**Published:** 2021-07-15

**Authors:** Ana Maria Linares, Jason M. Unrine, Amanda Thaxton Wigging, Juan C. Tantalean, Vlad C. Radulescu

**Affiliations:** ^1^College of Nursing, 751 Rose St, Room 435-A, CON Building, University of Kentucky, Lexington, KY 40536, USA; ^2^Environmental Toxicology, Department of Plant and Soil Sciences, Agriculture Science Center North, 1100 S. Limestone St. Room N-122T, University of Kentucky, Lexington, KY 40546, USA; ^3^College of Nursing, 751 Rose St, University of Kentucky, Lexington, KY 40536, USA; ^4^University San Luis Gonzaga de Ica, Director Bioslab EIRL, Calle Fermin Tanguis 150, San Miguel, Ica, Peru; ^5^Pediatric Hematology-Oncology, Hemophilia Treatment Center, Kentucky Children Hospital, 800 Rose St, Suite C 400, University of Kentucky, Lexington, KY 40536, USA

## Abstract

This exploratory, descriptive cohort study (*N* = 60) determined lead (Pb) and arsenic (As) blood concentrations in Peruvian children and their association with hematological parameters of iron-deficient anemia (IDA) and anthropometric measurement. The mean age of children was 10.8 months (SD = 4.7) and ranged from 3 to 24 months old. Anemia (Hb levels below 10.5 g/dL) was found in 20% of this cohort. Additionally, microcytosis (MCV < 70 fL) was present in 54%, and hypochromia (MCH < 23 pg) in 42% of the group of children. Chi-square analysis showed that 88% of the children with anemia also had microcytosis and hypochromia (*p* < 0.001). Pb and As were detected in 100% of the infants' blood samples, and the concentrations were significantly higher in older infants than in younger ones. Pb and As were not associated with the sex, anthropomorphic parameters, or infant hemogram changes. Infants who received iron supplementation were 87% less likely to have low Hb compared with those who did not (OR = 0.13, 95% CI = 0.02–0.88, *p*=0.04). Herbal tea intake was significantly associated with microcytosis and hypochromia. Our finding uncovered that hematological parameters for anemia are modified in Peruvian children with high levels of microcytosis and hypochromia. Concentrations of Pb and As were above method detection limits in all Peruvian children, but these were not associated with IDA or anthropometric measurements. A large study, including other variables, would benefit from allowing a more complex model predicting anemia in Peruvian children.

## 1. Introduction

Prevention of anemia in children younger than three years old is critical because anemia has a strong association with neurobehavioral and neurodevelopment delay that continues until adulthood [1, 2]. Iron-deficient anemia (IDA) limits psychomotor development in children. Despite the correction of anemia, children with IDA experienced poorer long-term performance in cognitive, social, and emotional functions that continue until adulthood [1–3]. In children under three years old, iron (Fe) is essential for proper brain myelination, neurogenesis, and brain cells differentiation [3]. Iron deficiency typically manifests with anemia (low hemoglobin, Hb), hypochromia (low mean corpuscular hemoglobin, MCH), microcytosis (low mean corpuscular volume, MCV), and anisocytosis (variation in red blood cell size reflected in a high red cell distribution width, RDW).

Most patients with IDA are treated effectively with oral Fe preparations; however, high concentrations of some potentially toxic trace elements in the body may interfere with Fe absorption and/or Fe integration in the hemoglobin. IDA in children has been linked to lead/arsenic (Pb/As) contamination [4–7]. Children with elevated lead (Pb) exposure may develop IDA since Pb and Fe use the same gastrointestinal tract transporter, leading to competitive interference of Fe absorption as well as interfering with several enzymatic steps in the heme synthesis pathway [4, 8–10]. Moreover, animal and human studies have confirmed that IDA increases intestinal absorption of Pb, which puts children at higher health risk [1, 3].

One of every two children under three years of age in Peru has IDA [11]. Based on a UNICEF report (2008), Peru has made critical steps toward increasing norms, policies, and programs that promote and protect children's health and rights [12]. However, because Peru is a country with great cultural, ethnic, linguistic, socioeconomic, and geographic diversity, the national averages tend to hide health disparities affecting many children [12]. Studies have shown that Peruvian children have one of the highest IDA rates globally [13–15], with prevalence in endemic areas reaching as high as 76% in Puno and 51% in Cusco [11].

Most Peruvian children are regularly screened for IDA after six months old and treated with oral Fe supplements when blood Hb below the normal values for age is detected [16]. However, the rate of anemia in Peru has remained steady in the last five years despite the government's efforts to reduce it with Fe supplementation program [11, 17].

Evidence about factors associated with IDA in Peruvian children is scarce. One publication reported that anemia in Peru is linked to sociodemographic characteristics such as living outside Lima and Callao, low socioeconomic households, and adolescent mothers [17]. Additionally, other studies described anemia associated with parasitic infestations [18], vitamin deficiency, and nutritional anemia [19]. To the best of our knowledge, no study has been conducted on the potential association between IDA and toxic trace-element exposure in Peruvian children, even though some evidence has shown a high level of contamination in Peruvian water and soil [20, 21]. This study aimed to identify Pb and As concentrations in children's blood and their association with IDA hematological parameters in a Peruvian children sample. Secondary, we evaluated anthropometric indices and potential association with hematologic values and potentially toxic trace elements.

## 2. Methods

This exploratory, descriptive cohort study determined toxic trace-elements blood concentrations in Peruvian children and their association with hematological parameters of IDA. The Ethical Medical Committee of the University of San Luis Gonzaga-Ica, Peru, granted IRB approval # 001-2018.

### 2.1. Setting

Participants were recruited from four local primary health care outpatient clinics that provide care in the Ica city, Peru. Data were collected in a single encounter in the clinic following a mother/infant visit to their health provider. Recruitment and data collection were conducted during May and June 2019. Inclusion criteria included: mother with a child between 3 to 24 months old, an infant apparently healthy at recruitment, and no known congenital disease. All families granted informed consent. Participants were compensated with $50 Peruvian soles (around $15 US dollars) for their participation. All participants with anemia received adequate treatments.

### 2.2. Sampling

Given the exploratory nature of this study, no a priori power analysis was conducted. However, a sample size of *N* = 60 was established considering the time available for the research and recruiting participants' feasibility. Sixty mothers with their children agreed to participate in the study, and the biospecimen collection was completed in *n* = 41 children. Unfortunately, nineteen children missed the blood sample collection at the time scheduled in the clinic. Efforts to reschedule the blood collection were unsuccessful.

### 2.3. Procedural

Nurses collected survey data from the mothers (one-time point), including sociodemographic characteristics, history and type of infant's feeding, and environmental and housing conditions. After completing the survey, infant anthropometric measurements and the blood sample were collected following standardized protocols. All samples were identified with the numerical code assigned to the participant.

#### 2.3.1. Child Anthropometric Measures

Weight, height, and head circumference were measured using a calibrated scale, a calibrated stadiometer, and a meter infant disposable paper tape measure. All measurements were completed in duplicate. We used the World Health Organization (WHO) [22] child growth standards for children between 0 to 24 months old to calculate age- and sex-specific z-score for height, weight, body mass index (BMI), and head circumference (HC). The use of z-scores allows for the comparison of anthropometric measurements at different ages at the time of recruitment.

*The infants' blood* was collected via venipuncture in two tubes: (1) Microtainer® MAP microtubes by Becton Dickinson (BD) and (2) Vacutainer EDTA tubes, Hemogard® by BD. Blood samples were transported on ice to a local lab. The local lab reported a complete blood count test (CBC), and the additional Vacutainer sample was stored at −20°C, waiting for shipping to the US.

#### 2.3.2. Analysis of Samples in the US

Samples were shipped by a courier company that ensured the appropriate temperature (at least −20°C) of blood samples at all shipping process stages. All samples were delivered to the University of Kentucky and were consigned and stored in the Clinical Center for Translational Science (CCTS) Lab and kept at −80°C. Frozen samples of the infants' blood were transferred to the Environmental Chemistry and Toxicology Lab, University of Kentucky, to analyze Pb and As concentrations.

The samples were digested in ultrapure nitric acid (Aristar Ultra; VWR) and analyzed to determine Pb and As concentrations using inductively coupled plasma mass spectrometry (Agilent 7500cx; Santa Clarita, CA, USA) based on previously validated methods [23]. Quality control procedures included analysis of procedural blanks, initial calibration verification, and analysis of standard reference materials (NIST SRM 955c, Toxic Metals in Caprine Blood; National Institute of Standards and Technology, Gaithersburg, MD, USA).

### 2.4. Data Analysis

Descriptive analysis was accomplished using means and standard deviations, median and interquartile range, or frequency distributions. We dichotomized the hematological parameters based on normal hematologic values in children (mean -2 SD) [24] to determine chronic medical conditions such as (a) anemia, Hb < 10.5 g/dL; (b) microcytosis, MCV < 70.0 fL; and (c) hypochromia, MCH < 23.0 pg [24]. Chi-square tests of association were used to evaluate associations among low hematological indices and the sex of the child. To evaluate associations among hematological values (anemia, microcytosis, and hypochromia) with age and anthropometric z-scores, the two-sample *t*-test was used; and the Mann–Whitney *U* test was used for association with levels of toxic trace elements in infant blood. Spearman's product correlation was used to determine whether trace elements correlate with children's age and anthropometric measurements. We used linear regression models to identify predictors of anthropometric z-scores. Finally, three logistic regression models were used to identify predictors of alteration of the hematological parameters listed above. All data analysis was conducted using SAS version 9.4, and we used a *p*-value of ≤0.05 to be statistically significant.

## 3. Results

Demographic characteristics are shown in Table 1. The mean age of children was 10.8 months (SD = 4.7) and ranged from 3 to 24 months. Most mothers had high school or GED education levels (64%) and have health insurance (70%), and household income was just enough to cover basic needs (62%). Most of the houses were solid construction (52%) with a concrete floor (66%). Current infant feeding status showed that most infants were fed with breast milk (88%), and 82% received complementary foods. More than half of the children (55%) received iron supplementation and got herbal teas (72%).

### 3.1. Hematological Parameters of Infant Blood

The CBC was reported in *n* = 41 samples of infants' blood (two samples were coagulated). Anemia (Hb levels < 10.5 g/dL) was found in 20% of this cohort. Additionally, microcytosis (MCV < 70.0 fL) was present in 54% and hypochromia (MCH < 23.0 pg) in 42% of the group of children (Table 1). There was a significant association between children with anemia and microcytosis (*p*=0.009) or hypochromia (88%, *p*=0.003). Moreover, we found that about half of the children have microcytosis and hypochromia without anemia. Additionally, microcytosis was significantly more common in females than males (79% vs. 43% respectively, *p* = 0.02, results not shown in tables). Age of the child was significantly (*p*=0.01) associated with microcytosis; children with microcytosis were older (mean 13.23, SD = 4.6 months) than children without microcytosis (mean 9.53, SD 4.6 months).

### 3.2. Infants' Anthropometric Characteristics

Children's average weight, height, BMI, and HC z-score calculated according to the WHO standards were 0.26 (SD = 1.22), −0.16 (SD = 1.33), 0.45 (SD = 1.23), and −1.29 (SD = 1.90), respectively (Table 1). There was no significant difference between groups of children with or without alteration of hematological levels (anemia, microcytosis, and hypochromia) and weight, height, and BMI z-scores. The weight z-score correlated positively with height, HC, and BMI z-scores (Table 2). Finally, child sex, age, and Pb and As concentrations were not associated (*p* > 0.05) with anthropometric z-scores in this sample of Peruvian children.

### 3.3. Infant Blood Trace-Elements Concentrations

Trace-elements analyses were conducted in 42 infants' blood samples. Pb and As were detected in 100% of the infants' blood samples. The median (interquartile range, IQR) concentration of Pb was 1.60 (IQR = 1.72) *μ*g/dL, while the median As concentration was 1.36 (IQR = 0.71) *μ*g/L (Table 1). Pb and As were positively and significantly (*p* < 0.05) associated with the age of the children (Table 2). However, Pb and As blood concentrations were not associated with hematological levels and the sex of the child.

### 3.4. Predictors of Anemia

We ran three logistic regression models to define predictors of anemia, microcytosis, and hypochromia (Table 3). First, the logistic regression modeling factors associated with low hemoglobin showed that the overall model was not significant (Chi-square = 6.7, *p*=0.08), even though iron supplementation was significantly associated with levels of Hb; infants who received iron supplementation were 87% less likely to have low hemoglobin compared with those who did not (OR = 0.13, 95% CI = 0.02–0.88, *p*=0.04). Pb and As blood concentrations were not related to low Hb status. We did not include herbal tea in this model because none of the babies with low Hb received herbal teas.

The second model showed predictors of microcytosis. The overall logistic model was significant (Chi-square = 10.42, *p*=0.03). Infants who were given herbal teas were significantly more likely to have microcytosis (OR = 13.34, 95% CI = 1.35–131.74, *p*=0.02) compared with those who did not drink herbal teas (Table 3). Finally, the hypochromia overall model was also significant (Chi-square = 10.55, *p*=0.03). Infants who were given herbal teas were significantly more likely to have hypochromia (OR = 13.04, 95% CI = 1.25–136.35, *p*=0.03) compared with those who did not drink herbal teas. Similar to the other models, Pb and As blood concentrations were not significant (Table 3).

## 4. Discussion

This study's primary aim was to analyze the association of Pb and As concentrations in infants' blood and their association with hematological parameters in Peruvian children. The levels of anemia detected in our sample based on Hb were lower (20%) compared with the Peruvian government levels (41%) in the area that we collected the samples [16]. The difference could be related to the testing methods used; while Peruvian clinics used a capillary sample to collect and analyze blood (HemoCue® Hb 201+) [25], we used a venipuncture blood sample, and the hemogram or CBC was analyzed by a Peruvian lab that followed international standards.

Results from HemoCue® Hb 201+ are highly correlated to those yielded by traditional CBC [26, 27], but differences in capillary sampling may produce systematic errors that influence results [28]. We also found that a significant percentage (88%) of children with anemia also had microcytosis and hypochromia. It is essential to notice that Peruvian infants are only tested via a capillary method without a hemogram's follow-up when the Hb result is low. Lack of analysis of hemogram or CBC might hide if microcytosis or hypochromia are presently preventing a clear picture of the hematological alteration that Peruvian children have.

It is known that microcytosis and hypochromia are the hallmarks of IDA, but other conditions might produce microcytosis and hypochromia that may be confused with IDA [1, 29]. Our results showed that some Peruvian children in our sample had microcytosis and hypochromia without IDA; this has been associated with other conditions, including genetic alterations like the thalassemia trait and anemia of chronic disease (ACD) [1]. A study to evaluate other potential variables related to anemia, including genetic traits, is needed in Peruvian children to clarify why they are more likely to develop anemia in the early stage of life. We highly recommend that children positive for anemia based on the HemoCue® Hb 201+ should be followed up with a hemogram to allow identification of other hematological alterations that might define other causes of IDA.

Anthropometric mean z-scores were normal according to the WHO standards when we considered children from the four clinics. We found no association between z-scores and age, sex, hematological levels, and Pb or As concentrations. Similar results have been reported in a cohort of Canadian children. In addition, the researchers found no significant association between children's blood concentration of Pb and As with childhood BMI, weight, or height in boys or girls [30].

Our findings showed that 100% of this cohort of children had fairly low concentrations of Pb and As in their blood. Our data showed a significant association between age and concentrations of Pb and As; as the child gets older, concentrations of Pb and As in blood increase. Although trace-elements concentrations were low, it concerns that we found these elements in 100% of the blood samples. Thresholds of blood Pb levels that have been associated with neurodevelopmental effects have tended to decrease over the years as research accumulates. The CDC has reported that a Pb concentration of >5 *μ*g/dL is used to indicate possible adverse neurodevelopmental outcomes for children older than one year [31]. Additionally, other institutions reported that a Pb concentration in children of 1.40 *μ*g/dL and higher should be investigated [32]. In a cohort of Canadian children, the median Pb level in blood reported was 0.66 *μ*g/dL [30]. In our cohort of Peruvian children's blood samples, we found that the median concentration was 1.60 *μ*g/dL, which is more than double compared with Canadian children but under the threshold suggested by the CDC. Nevertheless, our data showed that the concentration of Pb was not significantly associated with hematological or anthropometric alterations.

Arsenic causes a neurodevelopmental effect and has been associated with deficits in intelligence and memory [6]. As concentration in Peruvian infants' blood was extremely low (1.36 *μ*g/L) compared with the acceptable threshold concentration of <13 *μ*g/L [33]. Still, Peruvian children's As concentrations were much higher than those reported for Canadian children (1.36 vs. 0.46 *μ*g/L, respectively) [30]. Differences in diet may explain why 100% of Peruvian children have As in their blood. It is known that the Peruvian diet is high in the consumption of rice and seafood, which contain As [34, 35]. However, As contained in seafood is principally in the organic form, which is less toxic [35]. As concentration was not associated with hematological or anthropometric alterations in this sample of Peruvian children, which is similar to results in Canadian children [30].

The final logistic regression model showed that children supplemented with Fe were significantly less likely to be anemic. This is a promising result considering that the Peruvian government is establishing an iron supplementation program for children after six months old [16]. Therefore, a revision of inequality among Peruvian children on getting iron supplementation is warranted.

Microcytosis was detected in 54% of Peruvian children, and it was associated with the use of herbal tea. Moreover, hypochromia was detected in 42% of children, and the model showed that it was also predicted by increased intake of herbal teas. Tea interferes with Fe absorption and can lead to IDA when consumed in large quantities [36, 37]. This finding uncovers the need to educate the mothers about the permissive effect of herbal tea intake on Fe absorption in young children.

### 4.1. Strengths and Limitations

The principal strength of this study is that it provides evidence that Pb and As are present in blood samples of Peruvian children, even when their concentrations were low compared with the threshold proposed by the CDC [33, 38] and were not associated with IDA. On the other hand, the study has limitations as the sample size; mainly, it limited the inclusion of other variables in the logistic regression models. Additionally, recruitment was limited to one location in the country, which restricted the generalization of the findings. The sample collection methodology limited our ability to perform direct measurements of iron, ferritin, transferrin saturation, or genetic testing for thalassemia. However, with the limited resources and time allocated to this study, we generated this first approach to detect potential trace-element exposure associated with IDA in Peruvian children. A large study, including other variables, would benefit from allowing a more complex model predicting anemia in Peruvian children.

## 5. Conclusion

Our finding uncovered that hematological parameters for anemia are modified in Peruvian children with high levels of microcytosis and hypochromia. This study showed that concentrations of Pb and As were detectable in all Peruvian children and were positively and significantly associated with the child's age; however, these toxic trace elements were not associated with IDA or anthropometric measurements. In addition, our findings showed that receiving Fe supplementation significantly decreased the risk of having anemia. Consumption of herbal tea was significantly associated with microcytosis and hypochromia in Peruvian children.

## Figures and Tables

**Table 1:** Descriptive statistics of study variables (*N* = 60).

	Mean (SD), *n* (%) or median (interquartile range, IQR)
Child age (months)	10.8 (4.7)

Outpatient clinic	
A	24 (40%)
B	17 (28%)
C	6 (10%)
D	13 (22%)

Mother education level	
Elementary school	8 (13%)
High School/GED	38 (64%)
Some college education	14 (23%)

Health insurance	
Yes	42 (70%)
No	18 (30%)

Household income is enough to cover	
Less than basic needs	18 (30%)
Only basic needs	37 (62%)
More than basic needs	5 (8%)

Construction of house	
Clay	2 (3%)
Wood	17 (28%)
Cement-solid	31 (52%)
Other	10 (17%)

Floor type	
Soil-sand	16 (27%)
Cardboard	1 (2%)
Cement-solid	40 (66%)
Tile	3 (5%)

Sex of the child	
Female	25 (42%)
Male	20 (33%)
Unknown	15 (25%)

Feeding with breast milk	
Yes	53 (88%)
No	7 (12%)

Complementary foods	
Yes	49 (82%)
No	11 (18%)

Infant iron supplementation	
Yes	33 (55%)
No	27 (45%)

Infant drink herbal teas	
Yes	43 (72%)
No	17 (28%)

Infant anemia by age Hb < 10.5 g/dL^a^	
Yes	8 (20%)
No	33 (80%)

Infant microcytosis MCV < 70.0 fL^a^	
Yes	22 (54%)
No	19 (46%)

Infant hypochromia MCH < 23.0 pg^a^	
Yes	17 (42%)
No	24 (58%)

Infant weight z-score^b^	0.26 (1.22)

Infant height z-score^b^	−0.16 (1.33)

Infant BMI z-score^b^	0.45 (1.23)

Infant head circumference z-score^c^	−1.29 (1.90)

Infant blood lead concentration (*μ*g/dL)^d^	1.60 (IQR 1.72)

Infant blood arsenic concentration (*μ*g/L)^d^	1.36 (IQR 0.71)

^a^ *n* = 41; ^b^*n* = 44; ^c^*n* = 43; ^d^*n* = 42.

**Table 2:** Correlation of infants' age, blood toxic elements, and anthropometric measurements (*n* = 42).

Variable	1	2	3	4	5	6
1. Age	—					
2. As	0.37^*∗*^	—				
3. Pb	0.35^*∗*^	0.26	—			
4. Weight z-score	−0.9	−0.04	−0.06	—		
5. Height z-score	−0.23	−0.04	−0.00	0.45^∗∗^	—	
6. HC z-score	−0.17	−0.25	−0.03	0.32^*∗*^	0.24	—
7. BMI z-score	−0.12	−0.03	−0.10	0.82^∗∗^	0.11	0.33^*∗*^

^*∗*^ *p* < 0.05; ^∗∗^*p* < 0.01.

**Table 3:** Logistic regression examining predictors of hematological (low hemoglobin, microcytosis, and hypochromia) parameters associated with Pb and As (*n* = 40)^*∗*^.

	Low hemoglobin			Microcytosis			Hypochromia		
	OR	95% CI for OR	*p*	OR	95% CI for OR	*p*	OR	95% CI for OR	*p*
Iron supplementation									
Yes No	0.13 Ref	0.02–0.88	0.04	1.39 Ref	0.16–3.26	0.67	0.83 Ref	0.18–3.78	0.81
Herbal teas^a^									
Yes No	—	—	—	13.34 Ref	1.35–131.74	0.03	13.04 Ref	1.25–136.35	0.03
Blood lead concentration (*μ*g/dL)	1.27	0.62–2.12	0.44	1.35	0.70–2.61	0.36	1.27	0.68–2.37	0.45
Blood arsenic concentration (*μ*g/L)	0.30	0.03–2.78	0.29	0.41	0.07–2.17	0.29	0.20	0.03–1.23	0.08

^*∗*^2 blood samples were coagulated. ^a^Low hemoglobin not adjusted for herbal teas due to complete separation.

## Data Availability

The data are available by request to the first author.
